# Thiazoles and Thiazolidinones as COX/LOX Inhibitors

**DOI:** 10.3390/molecules23030685

**Published:** 2018-03-18

**Authors:** Konstantinos Liaras, Maria Fesatidou, Athina Geronikaki

**Affiliations:** Department of Pharmaceutical Chemistry, School of Pharmacy, Aristotle University, 54124 Thessaloniki, Greece; liarasn@gmail.com (K.L.); fesa.maria@gmail.com (M.F.)

**Keywords:** thiazole, thiazolidinone, COX, LOX, anti-inflammatory

## Abstract

Inflammation is a natural process that is connected to various conditions and disorders such as arthritis, psoriasis, cancer, infections, asthma, etc. Based on the fact that cyclooxygenase isoenzymes (COX-1, COX-2) are responsible for the production of prostaglandins that play an important role in inflammation, traditional treatment approaches include administration of non-steroidal anti-inflammatory drugs (NSAIDs), which act as selective or non-selective COX inhibitors. Almost all of them present a number of unwanted, often serious, side effects as a consequence of interference with the arachidonic acid cascade. In search for new drugs to avoid side effects, while maintaining high potency over inflammation, scientists turned their interest to the synthesis of dual COX/LOX inhibitors, which could provide numerous therapeutic advantages in terms of anti-inflammatory activity, improved gastric protection and safer cardiovascular profile compared to conventional NSAIDs. Τhiazole and thiazolidinone moieties can be found in numerous biologically active compounds of natural origin, as well as synthetic molecules that possess a wide range of pharmacological activities. This review focuses on the biological activity of several thiazole and thiazolidinone derivatives as COX-1/COX-2 and LOX inhibitors.

## 1. Introduction

Inflammation is a natural process that is connected to various conditions and disorders such as arthritis, psoriasis, cancer, infections, asthma, etc. Based on the fact that cyclooxygenase isoenzymes (COX-1, COX-2) are responsible for the production of prostaglandins that play an important role in inflammation, traditional treatment approaches include administration of nonsteroidal anti-inflammatory drugs (NSAIDs), which act as selective or non-selective COX inhibitors [1,2,3].

It is well established that COX-1 inhibitors, such as acetylsalicylic acid, induce gastrointestinal irritation, due to the fact that this particular COX isoenzyme is responsible for the production of gastroprotective prostaglandins. Moreover, this group of drugs can be responsible for increased bleeding diathesis resulted from inhibiting COX-1 catalyzed production of thromboxane A_2_ (TXA_2_). The severity of side effects caused by COX-1 or combined COX-1/COX-2 inhibitors (e.g., ibuprofen) concentrated the scientific interest on the production of selective COX-2 inhibitors, inspired by evidence supporting over-expression of this particular isoenzyme during inflammatory conditions. However, the hope for this new generation of drugs to be more effective than their predecessors and with less severe side effects was overshadowed by their association with increased myocardial infarction risk and cardiovascular thrombotic events. These severe side effects are mainly a result of inhibition of COX-2 catalyzed production of prostacyclin (PGI_2_), a prostaglandin possessing vasodilatory and antiaggregatory properties [4,5,6,7].

**Figure 1:**
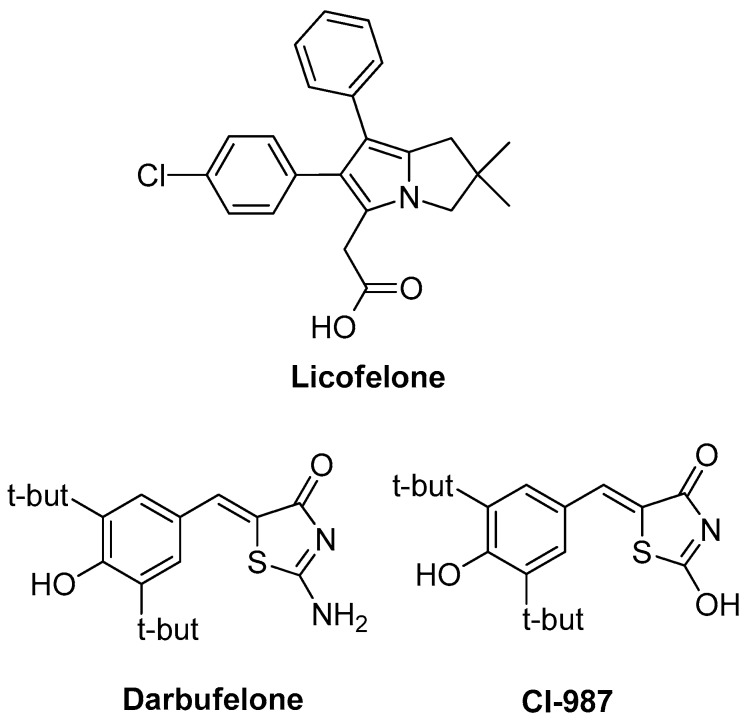
Chemical structures of Licofelone, Darbufelone and CI-987.

In search for new drugs to avoid side effects, while maintaining high potency over inflammation, scientists turned their interest on leukotrienes and lipoxins, which are produced via the lipoxygenase (LOX) pathway and are associated with various procedures such as leucocytes activation and adhesion to vascular endothelium, bronchial asthma pathogenesis, formation of edema and gastric mucosa damage [2,8,9]. Consequently, dual COX/LOX inhibitors could provide numerous therapeutic advantages in terms of anti-inflammatory activity, improved gastric protection and safer cardiovascular profile compared to conventional NSAIDs. Therefore, in the recent years, a notable research effort in the field of dual-acting COX/LOX inhibitors with very promising results has been observed [10,11]. One of the eminent compounds that was a result of the above-mentioned scientific effort is licofelone (Figure 1), a dual COX/5-LOX inhibitor that was under production by the pharmaceutical industry, with mixed results of phase III of clinical trials for osteoarthritis patients [12]. While being a non-selective COX inhibitor, it simultaneously inhibits 5-lipoxygenase activating protein (FLAP) [13]. It was proved that polypharmacological activity of licofelone is supported by inhibition of mPGES-1 [14]. Recently, it was found that licofelone modulates neuroinflammation in the chronic phase of spinal cord injury [15]. It is believed that this action is due to elevation of levels of endogenous anti-oxidants and anti-inflammatory metabolites in the lesion site. In another publication, the effect of licofelone in intracerebroventricular streptozotocin–induced cognitive deficit in rats was observed [16]. Two other compounds that are mentioned in the literature as dual COX/LOX inhibitors are thiazol-4(5*H*)-one derivatives: darbufelone and CI-987 (Figure 1) [17].

It is well established that compounds that contain sulfur atoms play a significant role in living organisms [18,19]. In particular, thiazole is a well-known heterocyclic aromatic compound that contains sulfur and nitrogen atoms at positions 1 and 3 of its five-member ring, respectively [20]. Τhiazole moiety can be found in numerous biologically active compounds of natural origin (e.g., thiamine [19,20], mycothiazole [21], cystothiazole C [22], as well as synthetic molecules possessing a wide range of pharmacological activities such as antimicrobial [23,24,25,26,27,28], antiviral [29,30], antitubercular [23,31], anti-inflammatory [32,33,34], anxiolytic [35], anaesthetic [36], anticonvulsant [37,38,39,40], etc.). There is a large number of known marketed drugs containing thiazole rings, such as the anthelmintic tiabendazole, the antibacterial sulfathiazole, the anticonvulsant riluzole, the anti-ulcer alizatidine, the antiparkinsonian talipexole, the antischistosomal niridazole, the antiviral ritonavir and the anti-inflammatory meloxicam.

Thiazolidinones, on the other hand, are derivatives of thiazolidine, a saturated form of thiazole, with a carbonyl group at position 2, 4, or 5. The chemistry of thiazolidinones has drawn scientific interest through the years because this particular ring system is the core structure in a variety of synthetic compounds with a broad spectrum of biological activities, such as antimycobacterial [41,42], antifungal [43,44,45,46], anti-cancer [47,48,49,50], anticonvulsant [51,52,53], anti-edematous [54], antidiarrheal [55], anti-HIV [56,57], anti-platelet-activating factor [58], antidiabetic [59], antihistaminic [60], anti-inflammatory [61,62,63], analgesic [64,65], antimicrobial [66,67,68,69], antidepressant [70], etc.

This review focuses on the biological activity of several thiazole and thiazolidinone derivatives as COX-1/COX-2 and LOX inhibitors. Literature references that are included in this review were found using mainly Google Scholar, Scopus and SciFinder (keywords: thiazoles COX, thiazoles LOX, thiazolidinones COX, thiazolidinones LOX, NSAIDs, inflammation, thiazoles activity, thiazolidinones activity, etc.).

## 2. Thiazoles as COX/LOX Inhibitors

**Figure 2:**
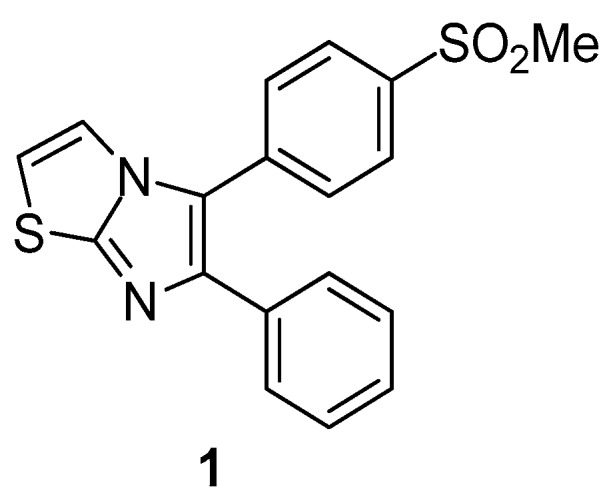
Chemical structure of 5,6-diarylimidazo[2.1-b]thiazole derivative 1.

Therien et al. [71] reported the synthesis of a series of 5,6-diarylimidazo[2.1-b]thiazole derivatives and evaluated their possible inhibitory potential against COX-2 and COX-1 enzymes. As a result, compound **1** (Figure 2) was identified as a potent, orally active and selective inhibitor of the COX-2 enzyme. This result was confirmed by *in vivo* evaluation of anti-inflammatory activity.

**Figure 3:**
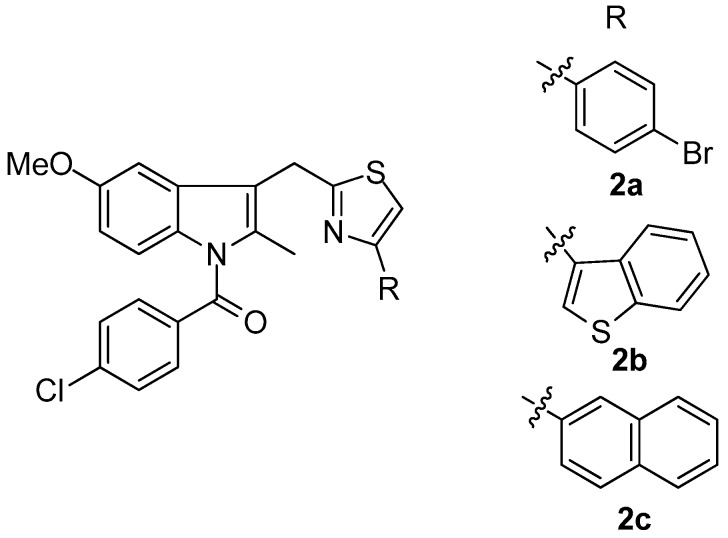
Chemical structures of 4-substituted thiazole analogues of indomethacin **2a**–**c**.

Woods et al. [72] synthesized a series of 4-substituted thiazole analogues of indomethacin, which were tested as inhibitors of COX-1 and COX-2. It was found that compounds are selective inhibitors of COX-2 while only moderate COX-1 activity (<57% inhibition at 10 mM) was observed. The most active compounds as COX-2 inhibitors appeared to be **2a**–**c** (Figure 3) with IC_50_ values of 0.3, 1 and 7 nM, respectively.

**Figure 4:**
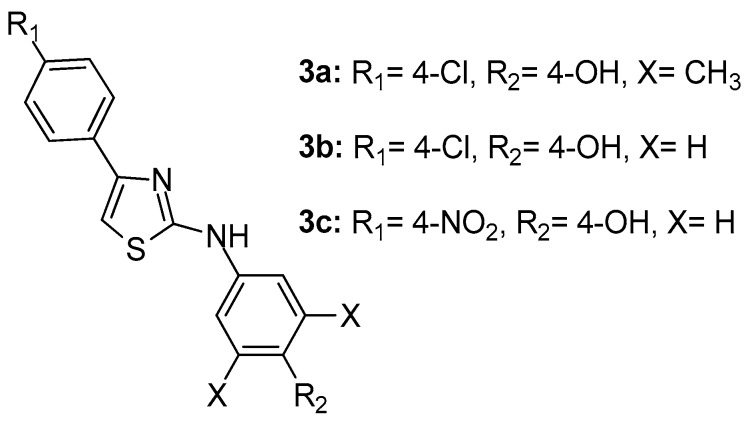
Chemical structures of N-aryl-4-aryl-1,3-thiazole-2-amine derivatives **3a**–**c**.

A series of N-aryl-4-aryl-1,3-thiazole-2-amine derivatives were synthesized by Suh et al. [73] as direct 5-LOX inhibitors. The SAR and chemical optimization studies revealed that, among 32 synthesized compounds, **3a**, N-(3,5-dimethylphenyl)-4-(4-chlorophenyl)-1,3-thiazole-2-amine (Figure 4), was the most potent LOX inhibitor with 98% inhibition (IC_50_ = 127 nM) and 98% inhibition in a cell-based assay. Compounds **3b** and **3c** (Figure 4), although possessing strong LOX inhibitory activity, with IC_50_ values of 35 and 25 nM respectively, cell-based assay results showed rather moderate potential.

**Figure 5:**
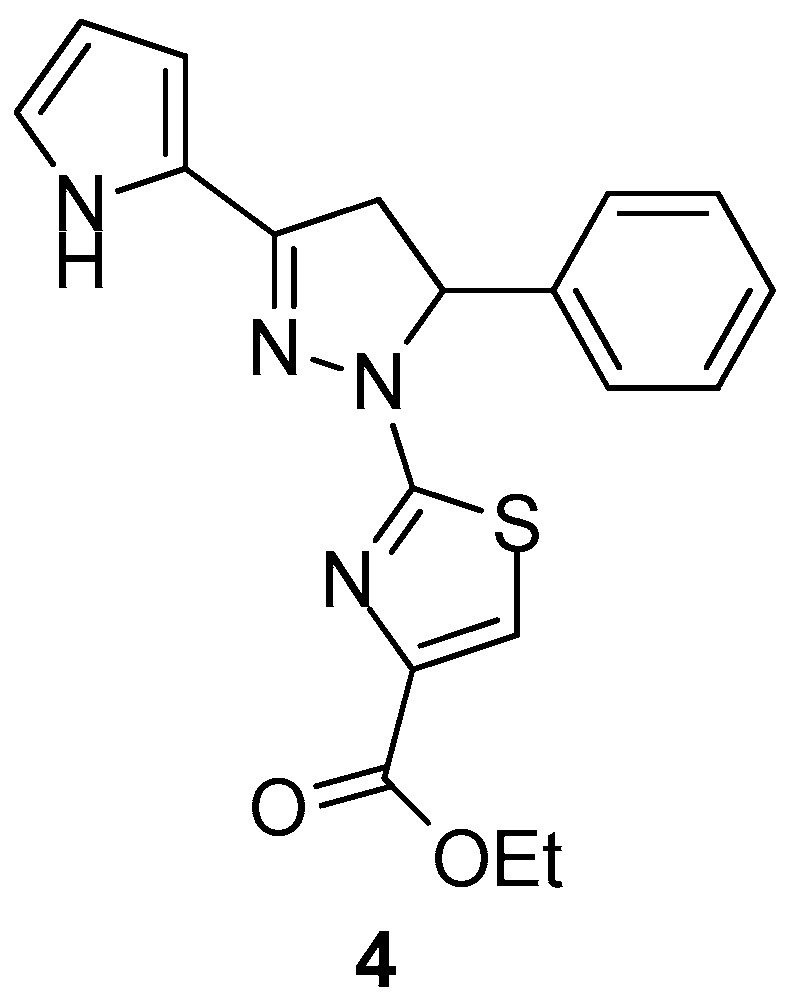
Chemical structure of 1-(4-ethylcarboxylate-thiazol-2-yl)-3,5-di(hetero)aryl-2-pyrazoline derivative **4**.

Carradori et al. [74] reported the synthesis of novel 1-(4-ethyl carboxylate-thiazol-2-yl)-3,5-di(hetero)aryl-2-pyrazoline derivatives as potential inhibitors of human COX isoenzymes. In vitro assay displayed promising selectivity against COX-1, with compound **4** (Figure 5) possessing the strongest activity with IC_50_ = 29.60 ± 1.58 μΜ, while none of the compounds exhibited COX-2 inhibition.

**Figure 6:**
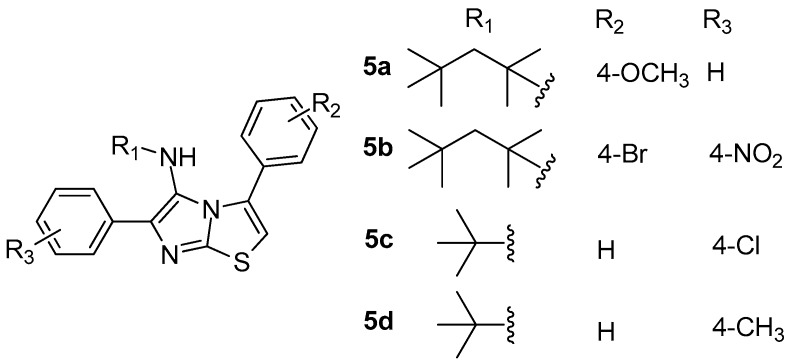
Chemical structures of 3,6-diphenylimidazo[2,1-b]thiazol-5-amine derivatives **5a**–**5d**.

As a continuation of their research on the development of 15-LOX inhibitors [75], a series of new 3,6-diphenylimidazo[2,1-b]thiazol-5-amine derivatives were designed, synthesized and evaluated as inhibitors of the above enzyme by Tehrani et al. [76]. The study revealed that, among 14 synthesized and tested derivatives, **5a**–**5d** (Figure 6) appeared to be the most potent with IC_50_ values ranging between 11.5–35 μM. Compound **5a**, with 2,4,4-trimethylpentan-2-yl pendent group, was the most active compound, being two times more potent than reference drug quercetin (IC_50_ = 23 μM).

According to docking studies, **5a** interacts properly with target enzyme 15-LOX, with hydrophobic interactions playing an important role in the binding process.

**Figure 7:**
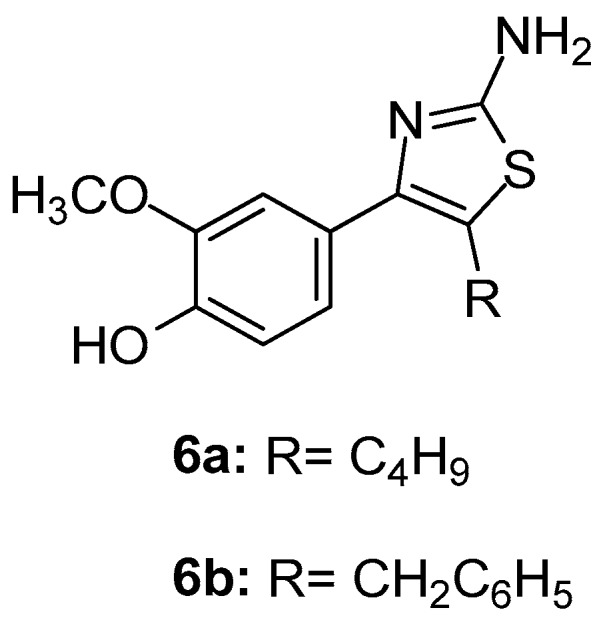
Chemical structures of compounds **6a** (N-[4-(4-hydroxy-3-methoxyphenyl)-1,3-thiazol.-2-yl]acetamide) and **6b** (4-(2-amino-1,3-thiazol-4-yl)-2-methoxyphenol).

Elachkar et al. [77] designed and synthesized two novel thiazole derivatives (Figure 7), namely compound **6a** (N-[4-(4-hydroxy-3-methoxyphenyl)-1,3-thiazol-2-yl]acetamide) and compound **6b** (4-(2-amino-1,3-thiazol-4-yl)-2-methoxyphenol), with aim to analyze their effect on COX isoforms. It was shown, using cell-stably over-expressing COX-1 and blood platelets, that compound **6a** was a non-selective COX-1/COX-2 inhibitor, while **6b** was a selective COX-2 inhibitor with similar IC_50_s (IC_50_s 9.01 ± 0.01 mM and 11.65 ± 6.20 mM). Furthermore, these compounds demonstrated anti-inflammatory activity according to the dorsal air pouch model of inflammation.

Docking studies revealed that both compounds **6a** and **6b** bind to the COX-2 active site in a similar manner as celecoxib.

**Figure 8:**
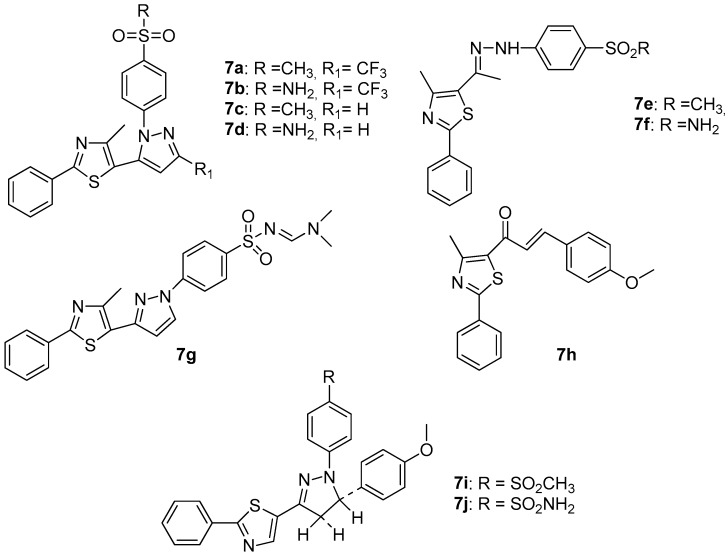
Structures of thiazolo-celecoxib analogues.

Abdelall et al. [17], by modification of the celecoxib molecule, designed and synthesized some thiazolo-celecoxib analogues (**7a**–**7j**, Figure 8) and evaluated their anti-inflammatory, COX-1, COX-2 and 15-LOX inhibitory activity.

The study of COX-1, COX-2 as well as 15-LOX inhibitory activity revealed that all compounds possessed COX-1, COX-2 and 15 –LOX inhibitory potency. Compounds **7a**, **7b**, **7e** and **7i** were the most active COX-1 inhibitors, with IC_50_ values of 4.80–6.30 μΜ being better than celecoxib, which was used as a reference drug (IC_50_ 7.60 μΜ), but not better than aspirin. The same compounds appeared to be very potent COX-2 inhibitors (IC_50_s 0.98–1.71 μΜ) better than aspirin, while compounds **7a**, **7b** and **7i** appeared to also be good 15-LOX inhibitors with IC_50_s of 3.98–5.41 μΜ, exhibiting higher potency than meclofenamate sodium that was used as a reference drug. Nevertheless, two compounds reached the goal of the authors. Compounds **7a** and **7i** possessed dual COX-2/15-LOX activity, despite their good COX-1 potency. This result proved the rationality of the authors’ design.

**Figure 9:**
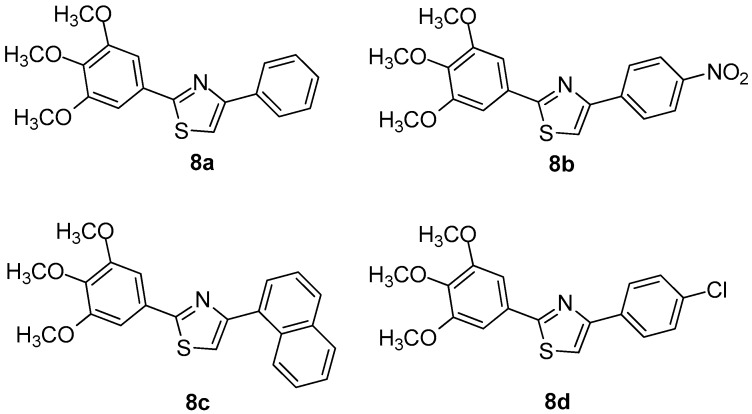
Chemical structures of compounds **8a**–**d**.

Oniga et al. [7] designed and synthesized a series of new 2-(trimethoxyphenyl)-thiazoles aiming to develop new, safer and less toxic compounds as NSAIDs. In order to elucidate their mechanism of action, the authors performed evaluation of their COX-1/COX-2 inhibitory potency. In addition, docking studies were performed. It was found that four (**8a**–**8d**) out of thirteen tested compounds were the most active (Figure 9), even though no compound exhibited COX-1/COX-2 activity higher than reference drugs. Docking studies revealed that compounds **8a** and **8c** occupied area close to that of meloxicam, forming hydrogen bonds with the key amino acids Arg120, Ser530 of the active site of COX-2 enzyme. The similar behavior, regarding a COX-1 active site, was observed for the above-mentioned compounds. According to the authors, this is due to the homology similarity of active sites of two enzymes. The SAR studies revealed that substitution in position 4 of the phenyl ring is very important for the selectivity towards the COX-2 enzyme.

**Figure 10:**
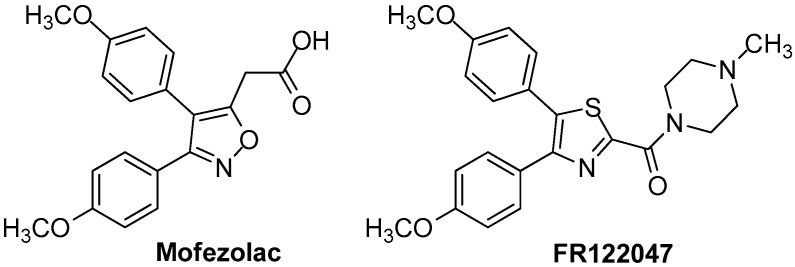
Chemical structures of COX inhibitors **Mofezolac** and **FR122047**.

Novel 4,5-diarylthiazoles COX-1 inhibitors were reported by Abdelazeem and co-workers [78]. These compounds were designed and synthesized in order to be tested as COX inhibitors analogously to mofezolac and FR122047 (Figure 10), which lack a gastric damaging profile.

**Figure 11:**
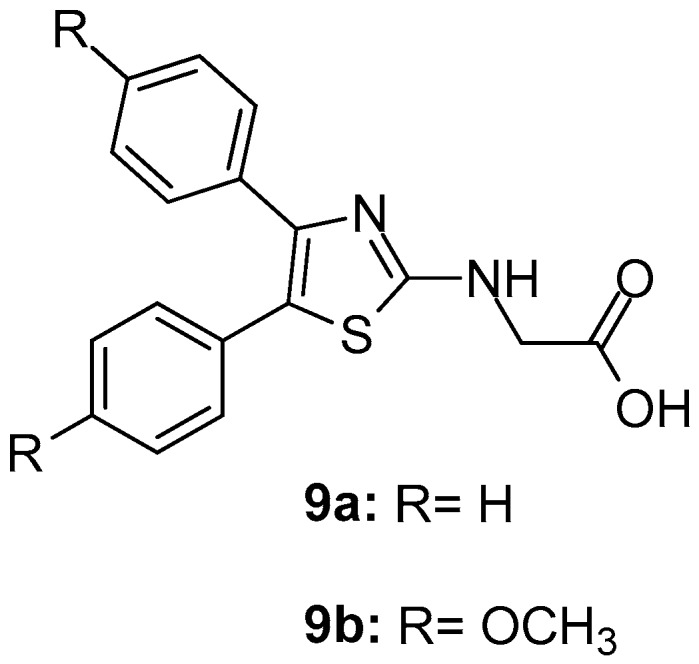
Chemical structures of 4,5-diarylthiazoles **9a** and **9b**.

These compounds were also evaluated for anti-inflammatory and analgesic activities. This study revealed that two compounds, **9a** and **9b** (Figure 11) were the most potent COX-1 inhibitors with IC_50_ values of 0.42 and 0.32 μM and moderate COX-2 inhibitors with IC_50_s 10.71 and 9.23 μM, respectively. The studies concerning ulcerogenicity revealed a significantly tolerable gastric profile. 

As a continuation of their previous research [78], Abdelazeem et al. [79] synthesized a series of novel diphenyl thiazole derivatives aiming to evaluate their anticancer activity.

**Figure 12:**
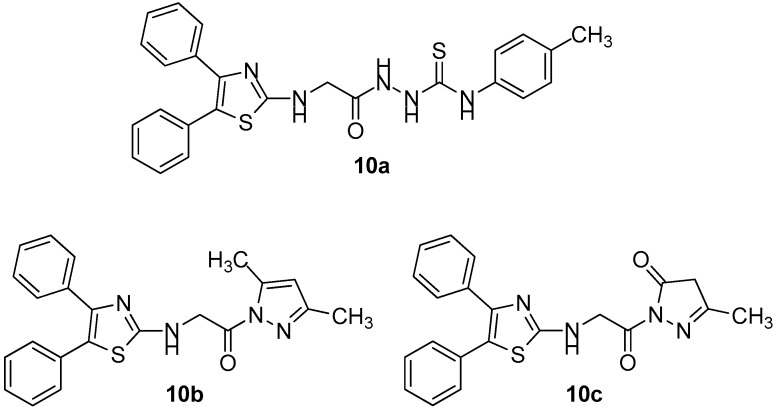
Chemical structures of diphenyl thiazole derivatives **10a**–**10c**.

**Figure 13:**
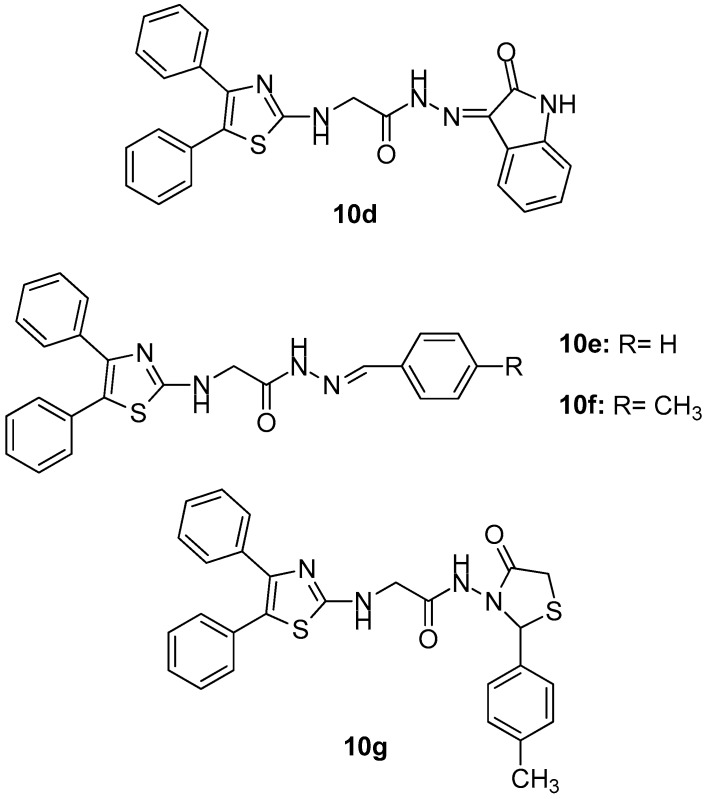
Chemical structures of diphenyl thiazole derivatives **10d**–**10g**.

Taking into account the growing interest of scientific community in studying the potential anticancer activity of COX-2 inhibitors, the authors evaluated anti-inflammatory and also COX inhibitory activity of compounds **10a**–**10g** (Figure 12 and Figure 13) that possessed the best anticancer profile against a panel of cancer cell lines (MCF-7, HT-29, A549).

The most active as COX-1 inhibitor was **10b** (IC_50_ = 4.8 μM), while **10f**, the most active anticancer compound against previously mentioned cell lines (with IC_50_ = 0.96 μM as COX-2 inhibitor) showed the highest selectivity index concerning this particular isoenzyme compared to diclofenac. Based on the fact that all tested compounds showed a good COX-2 selectivity, the authors came to the conclusion that there might be an important correlation between cancer treatment and the inhibition of the above-mentioned COX isoform.

## 3. Thiazolidinones as COX/LOX Inhibitors

**Figure 14:**
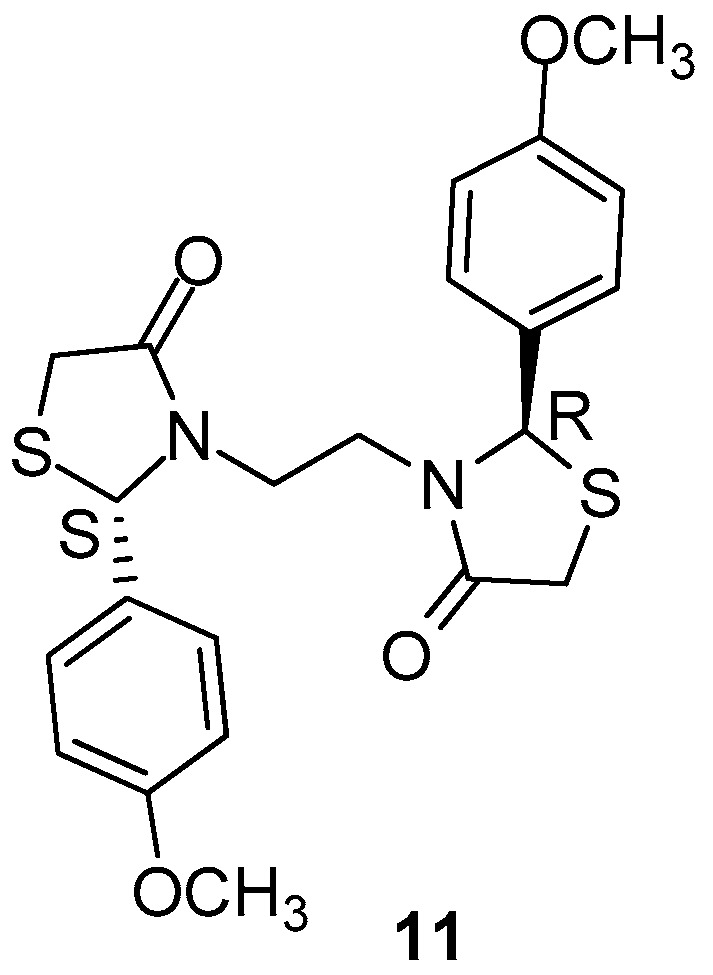
Chemical structure of 4-thiazolidinone derivative **11**.

Ottana et al. [80], with the aim to ameliorate the activity of lead compound (2*R*,2′*S*)3,3′-(1,2-ethanediyl)bis(2-(3,4-dimethoxyphenyl)-thiazolidin-4-one [81], made some modifications such as removal of 3-methoxygroups of the benzene ring, retaining the 4-methoxy groups. The synthesized compound **11** (Figure 14) was screened for its anti-inflammatory activity as well as for its gastrointestinal safety [82]. The authors evaluated the new compound, after corresponding modifications, for its possible COX-1/COX-2 inhibitory activity. It was found that the novel compound is a better COX-2 inhibitor than the lead compound of this class, as it was observed inthe human whole blood assay and computational studies. The selectivity index COX-1/COX-2 appeared to be more than 30 times higher than that of a previously mentioned lead compound. Based on the findings, the authors confirmed the well-established fact that overproduction of COX-2 derived prostaglandins play a role in acute inflammation.

**Figure 15:**
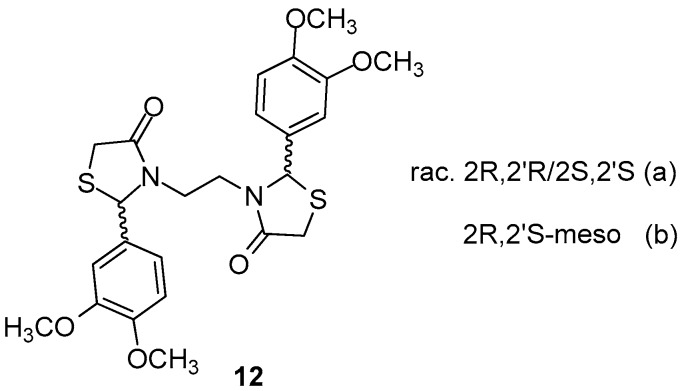
Chemical structures of racemic mixtures (a) and mesomeric (b) forms of 3,3′-(1,2-ethanediyl)-bis[2-(3,4-dimethoxyphenyl)-4-thiazolidinone 12.

Vigorita et al. [83] performed conformational studies as well as *in situ* molecular dynamics on previously synthesized [81] 3,3′-(1,2-ethanediyl)-bis[2-(3,4-dimethoxyphenyl)-4-thiazolidinones 12 (Figure 15), which were obtained as racemic mixtures (a) and mesomeric (b) forms *RR*, *SS*, *RS*, in order to better understand the binding mode of these forms to the active center of the enzymes. It was found that the *SS* enantiomer exhibited the highest binding affinity score with interaction energy of −47.15 kcal/mol, while the *RR* enantiomer showed low affinity for both COX isoforms. The meso form *RS*, although able to interact with both enzymes, showed, however, higher affinity for COX-2 with interaction energy −46.88 kcal/mol.

The authors, after in vitro evaluation of COX-1/COX-2 inhibitory activity, concluded that affinity order towards COX-1/COX-2 is following the order SS > RS > RR, in agreement with the theoretical results as well as previous in vivo data [82].

Theoretical results indicated SS > RS > RR affinity order towards COX-2 isoenzyme, in agreement with in vitro and previous in vivo pharmacological results.

**Figure 16:**
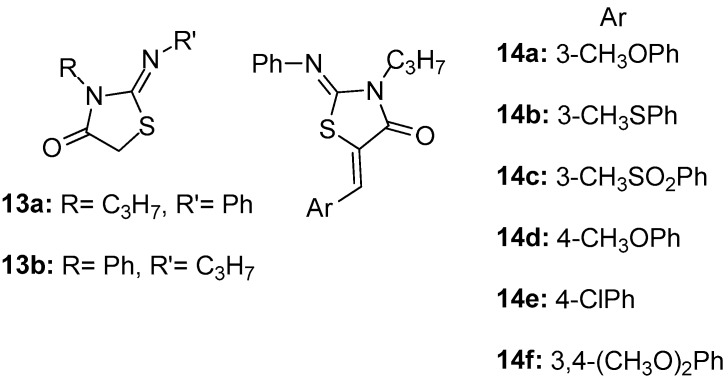
Chemical structures of 2-imino-4-thiazolidinone derivatives **13a**, **13b** and 5-arylidene-2-imino-4-thiazolidinone derivatives **14a**–**14f**.

As a continuation of their previous work [80], Ottana et al. [84] reported the design and synthesis of 2-imino-4-thiazolidinones **13a**, **13b** and 5-arylidene-2-imino-4-thiazolidinones **14a**–**14f** (Figure 16), which were evaluated for their in vivo anti-inflammatory activity by carrageenan-induced paw edema and pleurisy assays in rats [85,86]. Compound **14a**, 5-(3-methoxypnenyliden)-2-phenylimino-3-propyl-4-thiazolidine, exhibited very good anti-inflammatory activity in this assay. With the aim of investigating their possible mechanism of action, their ability to inhibit COX-1 and COX-2 was assessed in the murine monocyte/macrophage J774 cell line [87]. Finally, the most promising among the tested compounds, **14d**, was successfully docked into the active site of COX-2 enzyme, having as reference the known selective COX-2 inhibitor SC-558.

In this assay, compounds **13a** and **13b** exhibited only a weak inhibition of COX-1 isoform in all tested doses, without inhibiting COX-2. The introduction of a (*Z*)-5-arylidene group generally gave rise to the inhibition of COX-2 without reaching, however, the levels of the reference drugs.

**Figure 17:**
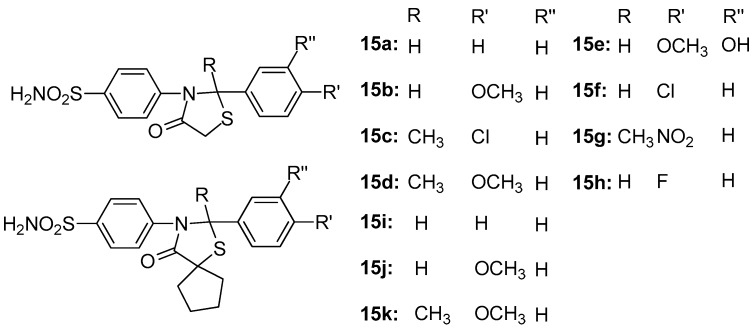
Chemical structures ofthiazolidine-4-one derivatives **15a**–**h**.

Taranalli et al. [88] synthesized 11 thiazolidine-4-one compounds, **15a**–**h** (Figure 17), and tested their anti-inflammatory activity in vitro (COX-1 and COX-2 inhibition) and in vivo (carrageenan-induced paw edema and cotton pellet-induced granulomas in rats). Most of the compounds showed significant inhibition of edema and granuloma dry weight, and, concerning in vitro COX-1 and COX-2 inhibition, half of the compounds tested showed maximum inhibition of COX-2, comparable to nimesulide. However, all tested compounds did not inhibit the COX-1 enzyme.

**Figure 18:**
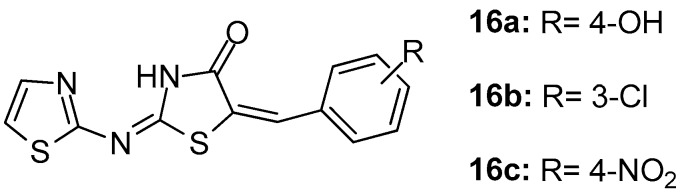
Chemical structures of 5-arylidene-4-thiazolidinones **16a**–**c**.

In their study, Geronikaki et al. [89] reported the computer aided design, synthesis and biological evaluation of nine novel 5-arylidene-4-thiazolidinone derivatives that were chosen as candidates out of 22 compounds predicted to be COX/5-LOX dual inhibitors. It was found that compounds **16a**, **16b** (Figure 18) were dual COX-1/LOX inhibitors (IC_50_s 158 μM/116 μM and 125 μM/125.9 μΜ, respectively), while compound **16c** appeared to be the COX-1 inhibitor (IC_50_ = 141 μM).

Hofmann and co-workers [90] reported the design, virtual screening for LOX inhibitory activity and synthesis of a series of 35 5-benzylidene-2-phenylthiazolinones. These compounds were evaluated in intact polymorphonuclear leukocytes (PMNL) and a cell-free assay.

**Figure 19:**
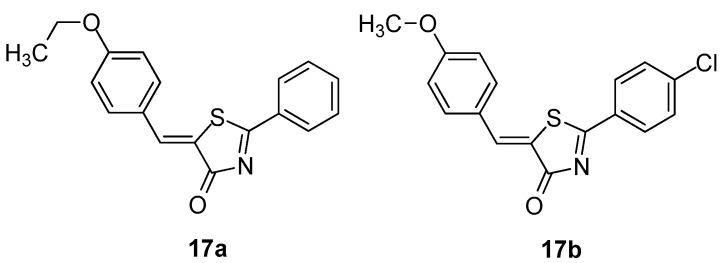
Chemical structures of 5-benzylidene-2-phenylthiazolinones **17a**, **17b**.

It was found that compound **17a** (Figure 19) caused potent inhibition of 5-LOX product formation in intact PMNL and in cell-free PMNL S100 with IC_50_ values of 2 and 0.5 μM, respectively. In order to improve activity, the authors made several modifications on the parent compound that led to several derivatives, among which the most potent was found to be compound **17b** (Figure 19), exhibiting the strongest LOX inhibitory activity with IC_50_s 0.09 μΜ and 0.28 μΜ in both assays (intact PMNL and in cell-free PMNL S100).

**Figure 20:**
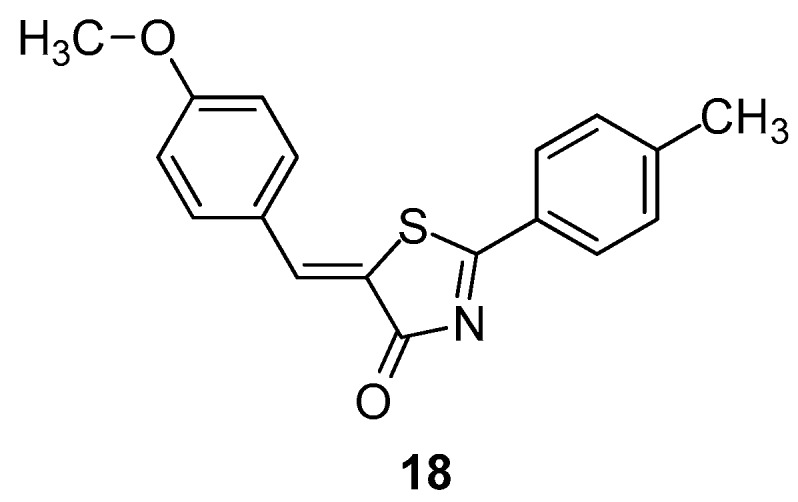
Chemical structure of 5-benzylidene-2-phenyl-5*H*-thiazol-4-one derivative **18**.

Hofmann et al. [91], taking into account their previous promising findings regarding ligand-based virtual screening of 5-benzylidene-2-phenyl-5*H*-thiazol-4-one derivatives [92] as LOX inhibitors, introduced structural modifications on compound **17a**. This led to the discovery of derivative **18** (Figure 20), which was investigated for its molecular pharmacological properties.

A pharmacological profile was studied both in a cell-based system using human PMNL and cell-free assays utilizing PMNL homogenates, S100 preparations of the homogenates and partially purified recombinant 5-LOX. It was found that derivative **18** seems to be a promising novel 5-LOX inhibitor with IC_50_ values in the nM concentrations range in intact cells and cell-free assays. It would be interesting to point out that compound **18** had a completely different mode of action compared to previously studied inhibitors of this class. Nevertheless, this compound appeared to be highly selective for 5-LOX.

**Figure 21:**
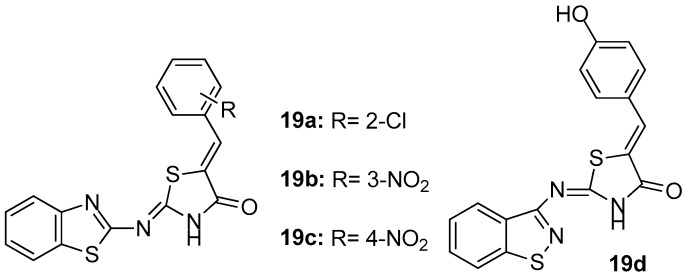
Chemical structures of benzothiazol-2-yliminothiazolidin-4-ones **19a**–**d**.

As a continuation of their previous work [89], Eleftheriou and co-workers [93], based on the knowledge that balanced modulation of several targets is crucial in the treatment of multifactorial diseases and knowing the inflammation mechanisms, proposed that balanced inhibition of COX-1/COX-2 and LOX enzymes could be a promising approach for the treatment of inflammation. Thus, a fragment–based library, focused on COX-1, COX-2 and LOX inhibition, by using chemoinformatics assays was created. As a result, 23 new benzothiazol-2-yliminothiazolidin-4-ones were designed and synthesized in order to evaluate their inhibitory activity on the above-mentioned enzymes. The evaluation of inhibitory activities revealed that most of the compounds showed potency against COX-1, with the best being compounds **19a**, **19b** and **19c** (Figure 21), with IC_50_ values of 0.018, 0.31 and 0.51 mM, respectively. Regarding COX-2, it was observed that the strongest inhibitory effect was exhibited by compound **19a** (58.8% inhibition), while, as far as LOX inhibition was concerned, almost all tested thiazolidinones were more potent than their thiazolyl analogues [89], having as a more potent compound **19d** with IC_50_ = 17.7 μΜ.

**Figure 22:**
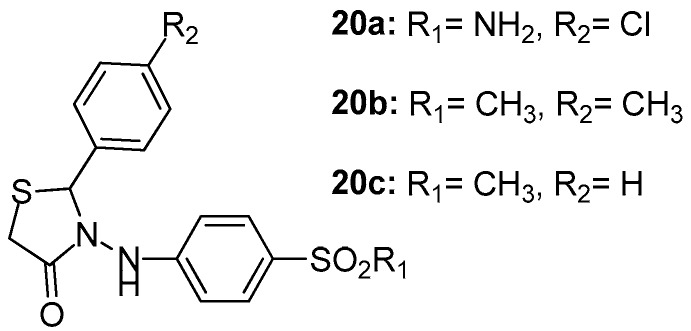
Chemical structures of 2-aryl-3-(4-sulfamoyl/methylsulfonylphenylamino)-4-thiazolidinones **20a**–**20c**.

Unsal-Tan et al. [94] reported the design of a series of novel 2-aryl-3-(4-sulfamoyl/methylsulfonylphenylamino)-4-thiazolidinones with the aim to develop new selective cyclooxygenase-2 inhibitors using molecular modeling studies by the MOE program. The designed thiazolidinone derivatives with reasonable binding modes and high docking scores were synthesized and evaluated for their COX-1/COX-2 inhibitory activities with NS-398 and indomethacine used as reference compounds. The activity of these thiazolidinones was relatively moderate against COX-1 enzyme, with the best activity demonstrated by compound **20a** (IC_50_ = 38.9 μΜ). On the other hand, COX-2 inhibitory activity appeared to be slightly better than COX-1, with the best activity shown by compound **20b** (IC_50_ = 14.4 μM) followed by compound **20c** (IC_50_ = 20 μM). The above-mentioned compounds are presented in Figure 22.

**Figure 23:**
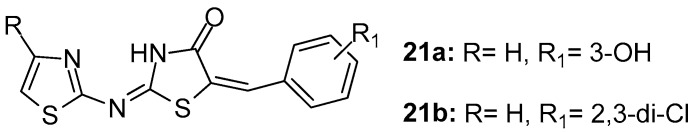
Chemical structures of 5-arylidene-2-(1,3-thiazol-2-ylimino)-1,3-thiazolidin-4-ones **21a**, **21b**.

Based on their previous studies [89], Apostolidis and co-workers [95] described the synthesis of novel 5-arylidene-2-(1,3-thiazol-2-ylimino)-1,3-thiazolidin-4-ones introducing different substituents in position 4 of thiazole ring and evaluated their anti-inflammatory, COX-1/COX-2 and LOX inhibitory activities. In general, all compounds showed moderate to low inhibitory activity against all three enzymes. Nevertheless, compounds **21a** and **21b** (Figure 23) demonstrated good activity against COX-1 with IC_50_s 16 and 10 μΜ, respectively.

Abdelazeem et al. [96] synthesized and evaluated the in vivo and in vitro anti-inflammatory activity of a series of diphenylthiazole–thiazolidinone hybrids. The evaluation of COX-1/COX-2 inhibitory potency revealed that, in general, all compounds demonstrated moderate to high activity. It was shown that the potency of compounds depends on the substituent on the thiazolidinone ring. Thus, replacement of five membered thiophene ring with pyridine or phenyl rings significantly increased the COX-1 inhibitory activity and selectivity.

**Figure 24:**
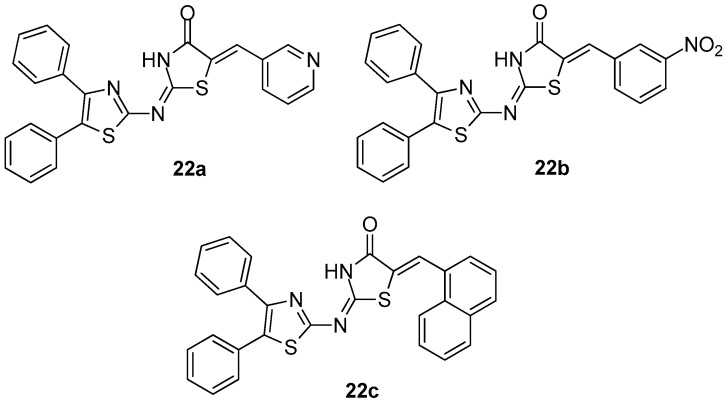
Chemical structures of diphenylthiazole–thiazolidinone hybrids **22a**–**c**.

Consequently, compounds **22a** and **22b** (Figure 24) appeared to be the most potent COX-1 inhibitors with IC_50_ values of 3.51 and 2.03 μΜ, respectively. The introduction of the bulky naphthyl group in position 5 of thiazolidinone ring resulted in compound **22c** (Figure 24) with strong COX-2 inhibition (IC_50_ = 3.84 μΜ). These in vitro results are in accordance with results of in vivo experiments and molecular docking.

**Figure 25:**
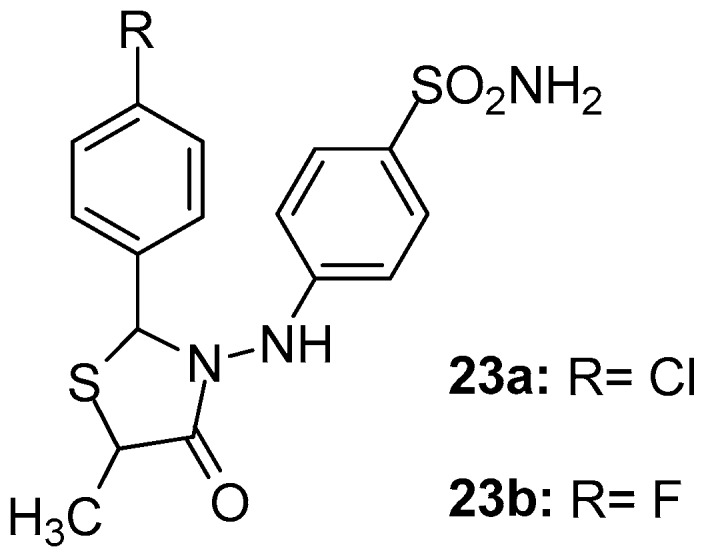
Chemical structures of thiazolidinone derivatives **23a**, **23b**.

As part of their ongoing studies on the synthesis of safe anti-inflammatory agents, Abdellatif et al. [97] designed and synthesized two series of new thiazolidin-4-ones as potential COX-2 selective inhibitors. Among the synthesized compounds, **23a** and **23b** (Figure 25) exhibited in general, the strongest in vitro COX-2 potential (IC_50_s 2.3 and 1.9 μΜ, respectively) and selectivity (SIs 4.56 and 5.68, respectively). Nevertheless, these two compounds showed good COX-1 inhibitory activity (IC_50_s 10.5 and 10.8 μΜ, respectively) as well. It should be noted that these results were in agreement with in vivo and molecular docking data.

**Figure 26:**
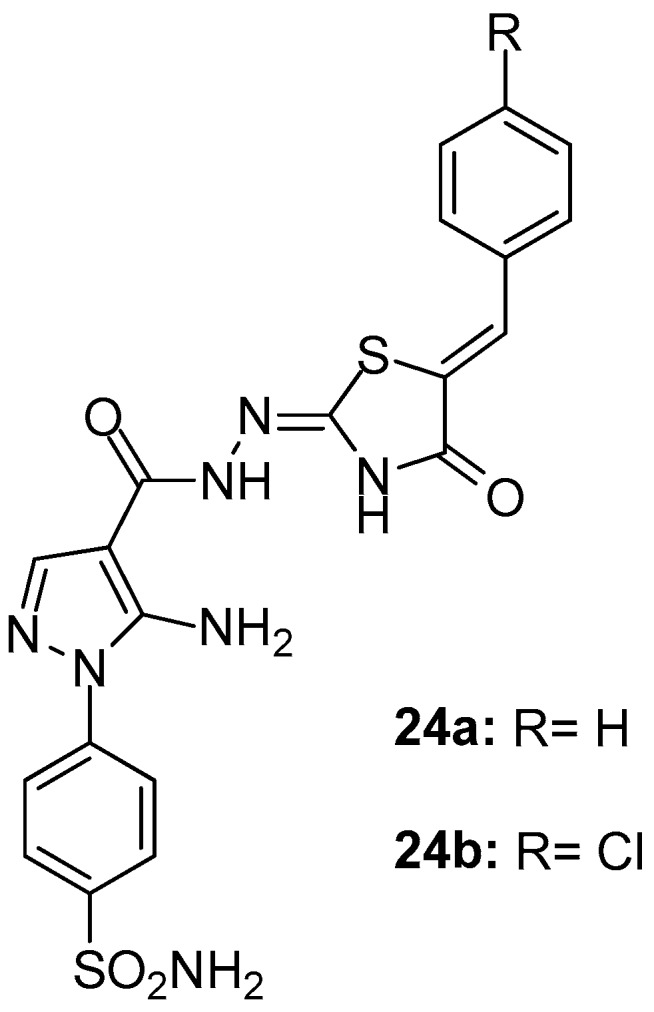
Chemical structures of pyrazolyl benzenesulfonamide derivatives **24a**, **24b**.

Ashour et al. [98] presented the synthesis and evaluation of in vitro/in vivo anti-inflammatory activity of new pyrazolyl benzenesulfonamides linked to polysubstituted pyrazoles and thiazolidinones. Among these two series of compounds, thiazolidinone derivatives and specifically compounds **24a** and **24b** (Figure 26) appeared to be the most potent COX-1/COX-2 inhibitors (IC_50_s 5.6/1.52 and 4.5/1.06 μΜ, SIs 3.68 and 4.24, respectively). It should be mentioned that, in general, the enzymatic inhibitory activity coincided with the results of the rat paw edema assay.

**Figure 27:**
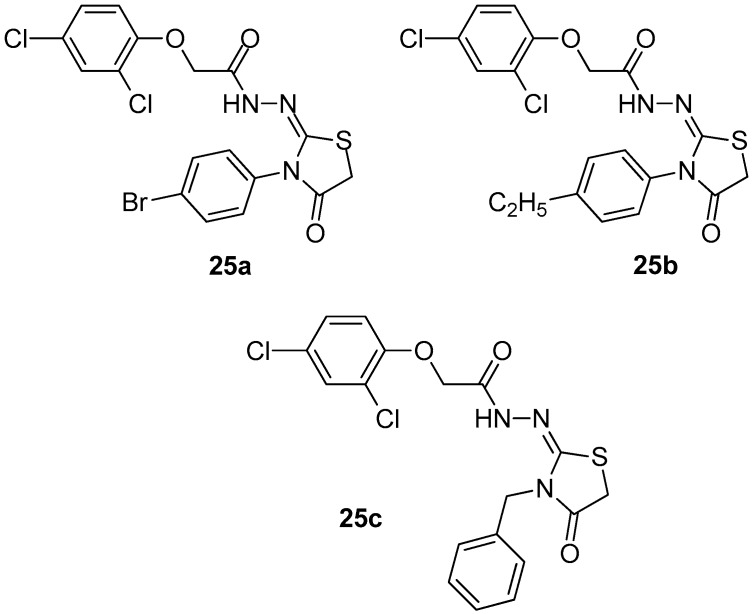
Chemical structures of 2-imino-4-thiazolidinone derivatives **25a**–**c**.

Fourteen 2-imino-4-thiazolidinone derivatives have been synthesized and evaluated by Ali et al. [99] for their in vivo anti-inflammatory activity. In order to elucidate the mechanism of action, docking on the COX-2 enzyme has been performed for all compounds. Based on docking results, three compounds, **25a**, **25b** and **25c** (Figure 27) were chosen for testing their COX-1/COX-2 inhibitory activity. The evaluation revealed that compound **25c**, with the highest anti-inflammatory activity and best glide energy among these three, appeared to also be the strongest COX-2 inhibitor with IC_50_ = 3.29 μΜ and SI = 29.00.

**Figure 28:**
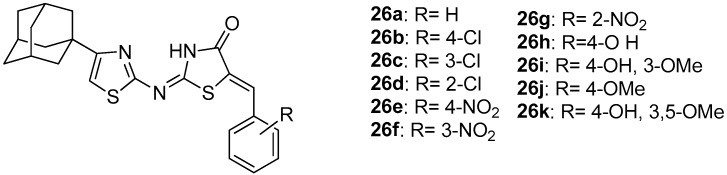
Chemical structures of 4-adamantanyl-2-thiazolylimino-5-arylidene-4-thiazolidinones.

Geronikaki et al. [100] used docking analysis to predict the effectiveness of new designed compounds (**26a**–**26k**, Figure 28) by insertion of adamantanyl moiety to previously synthesized and tested as COX/LOX inhibitors 2-thiazolylimino-5-arylidene-4-thiazolidinones [89]. It was found that compound **26c** had the best estimated binding energies to LOX and COX-2 (−12.57 kcal/mol and −12.54 kcal/mol), while the best estimated binding energy to COX-1 was observed for compound **26d** (−12.13 kcal/mol). It should be mentioned that, in all seven pairs of previously synthesized and new adamantanyl derivatives (**26a**–**26f** and **26i**), the estimated binding energy of the adamantanyl derivatives was lower than that of the non-substituted analogues for all three enzymes, showing a better predicted activity for the adamantanyl compounds.

Moreover, new adamantanyl derivatives, **26g** and **26h** were designed and evaluated using docking analysis. These two compounds did not have pairs with previous synthesized compounds. It was found that their predicted binding energies to COX and LOX enzymes was low, especially for compound **26g** (−12.22 kcal/mol). The evaluation of LOX inhibitory activity revealed that compounds **26c**–**26e**, **26g**, **26h** and **26k** exhibited good activity with IC_50_s 38, 98, 45, 34, 60 and 56 μM, respectively. As far as COX-1 inhibition is concerned, the best activity was found to possess compounds **26d** and **26b** (IC_50_s 39 μM and 50 μM respectively). Thus, experimental results coincide with docking, confirming the rational design of these thiazolidinone derivatives.

## 4. SAR

The analysis of the structure–activity relationship of thiazole derivatives used in this review revealed that thiazole based thiazolidinones were moderate to good COX-1/LOX inhibitors. The most favorable substituents in benzene ring appeared to be 4-NO_2_, 3-NO_2_ and 3-Cl. Introduction of a methyl group in positions 4 and 5 of thiazole rings led to compounds with only COX-1 inhibitory activity. LOX activity was lost. The introduction of adamantanyl substituent in position 4 of thiazole ring showed good LOX inhibitory activity. Furthermore, the presence of 2-NO_2,_ 4-NO_2_, 3-Cl, 4-Cl and 4-OH-3,5-OMe substituents in benzene rings increased LOX inhibition compared to unsubstituted rings. Introduction of 2 phenyl rings in positions 4,5 of thiazole rings led to COX-1/COX-2 inhibitors with the prevalence of COX-2 inhibition. The best activity against COX-2 was observed for compounds with bulky naphthalene group. Thiazolidinone derivatives with hydrazinocarbonyl pyrazolo benzensulfonamide group led to compounds with good COX-2 and moderate COX-1 potency. Introduction of phenylin position 4 of thiazolidin-4-one as well as benzensulfonamide group linked to nitrogen of the ring resulted in compounds with good COX-2 and moderate COX-1 activity. Linkage of two 3,4-dimethoxyphenylthiazolidine-4-one molecules together through the ethane link resulted in good COX-2 inhibitors with a high COX-1/COX-2 selectivity index.

The benzothiazole based thiazolidinones were much better as COX-1 inhibitors compared to thiazole-based thiazolidinones as well as better LOX inhibitors. The most favorable substituents on the benzene ring for COX-1 inhibition were 2-Cl, 4-Cl and 4-NO_2,_, while, for LOX, the substituents that improved activity were 2-NO_2_, 3-OH, 4-OH, 4-OMe, 4-OH, 3-OMe and 4-OH, 3,5-OMe. Replacement of the benzothiazole ring with benzoisothiazole resulted in less active compounds as COX-1 inhibitors, but substituents 2-NO_2_, 4-NO_2,_ 4-Cl, 4-OH and 4-OH-3-OMe substantially increased the LOX activity.

It was observed that, in general, the hybride molecules with thiazole and other heterocycles with bulky and lipophilic substituents expressed good COX-2 inhibitory potency. The same good activity resulted from a combination of thiazole with celecoxib, as well as phenyl substituted fused thiazole derivatives.

## 5. Conclusions

Over recent decades, much effort was dedicated to the development of new COX/LOX inhibitors. Specifically, there is a significant amount of publications concerning the synthesis and evaluation of thiazoles and thiazolidinones with potential COX/LOX inhibitory activity. Nevertheless, although a great amount of synthesized compounds were found to be potent as COX/LOX inhibitors, only a portion of them revealed a safer pharmacological profile, compared to marketed anti-inflammatory drugs. However, from the above-mentioned studies, it is clear that thiazole and thiazolidinone derivatives can be promising targets for future research in the field of COX/LOX inhibitors in order to discover new, more effective and safer anti-inflammatory drugs.
